# Prevalence of Antimicrobial-Resistant Bacterial Pathogens Among Livestock in Subtropical Environments

**DOI:** 10.3390/antibiotics15050461

**Published:** 2026-05-02

**Authors:** Benazir Kanwal, Ehtisham Asif, Nazeer H. Kalhoro, Urooj Zafar, Hassan Adil, Aqeel Ahmad

**Affiliations:** 1 Sindh Institute of Animal Health, Livestock & Fisheries Department, Government of Sindh, Karachi 74900, Pakistan; kanwalbenazir@gmail.com (B.K.); drnazeerhussain@hotmail.com (N.H.K.); 2 Department of Biotechnology, University of Karachi, University Road, Karachi 75270, Pakistan; ehtishamasif_89@yahoo.com; 3 Department of Microbiology, University of Karachi, University Road, Karachi 75270, Pakistan; aahmed57@gmail.com; 4 Diagnostic Division, U.M. Enterprises, Plot 12, Sector 15 Korangi Industrial Area, Karachi 74900, Pakistan

**Keywords:** antimicrobial resistance, Sindh institute of animal health, livestock, antibiotics

## Abstract

**Background/Objectives**: The prevalence of resistant pathogens in livestock and the environment threatens human health. Frequently used antibiotics in livestock gradually increase the resistance pattern, which intimately threatens the livestock industry. **Methods**: About 536 pathological samples were collected from January 2023 to December 2024 from chicken (472), birds (2), goat (25), sheep (12), and cows (21) and buffaloes (4), across the Sindh, Pakistan. A wide variety and number of bacterial pathogens were isolated and identified. After performing their antibiogram study, phylogenetic analysis was also performed. **Results**: The prevalence of different bacterial infections was studied in livestock. *Salmonella* spp. was found to be the most common cause of bacterial infection in livestock (77.79%), followed by *Escherichia coli* (62.69%) and *Staphylococcus aureus* (8.02%). The highest number of *Salmonella* spp. and *S. aureus* isolates were found to be resistant to ampicillin (80.67% and 81.4%, respectively), while *E. coli* isolates were found to be resistant to amoxicillin (97.87%) majorly. MARI revealed that 79.7% of *Salmonella* spp., including all MDR strains (*n* = 332); 55.59% of *E. coli* (*n* = 336), and 88.37% of *S. aureus* (*n* = 43) had indexes greater than 0.2. 16S bacterial identification and phylogenic analysis were performed through molecular methods. **Conclusions**: AMR is one of the most considerable livestock health issues that also affect human health. The MARI indicate a high rate of antibiotic use and resistance in the isolated *Salmonella*, *E. coli*, and *S. aureus* require the urgent need for continuous antimicrobial susceptibility surveillance and control of antibiotic use in livestock.

## 1. Introduction

Global health is increasingly jeopardized by the growing prevalence of antimicrobial resistance among diverse microorganisms in both human and livestock sectors affecting the One Health worldwide [1]. The 2025 WHO Global Antimicrobial Resistance Surveillance System (GLASS) report underscores antimicrobial resistance as a major global health burden, revealing that one in six bacterial infections in 2023 were antibiotic-resistant, with resistance increasing in 40% of pathogen–antibiotic combinations since 2018—particularly in South-East Asia and the Eastern Mediterranean—and notably high resistance documented in *Escherichia coli* (42% to third-generation cephalosporins) and *Staphylococcus aureus* (35% methicillin-resistant isolates) [2].

Microorganisms acquire resistant genes and mobile genetic elements from neighbors (resistant strains) present in the same environment [3]. AMR is a complex problem; it requires coordinated effort amongst health sectors, government agencies, and different stakeholders to frame it within the One Health approach. There is considerable evidence suggesting the cross-species transfer of resistant microorganisms from livestock to humans, which poses a massive threat to One Health prospective [4]. One Health is a joint effort of different organizations to improve the health of people, animals, and ecosystems [5]. It can help to resolve various issues of different disease control, including appropriate prophylaxis, diagnosis, therapeutics, and developing strategies to monitor and manage the spread of pathogens in a community. Furthermore, the One Health approach also focuses on the surveillance of the emergence and re-emergence of pathogens with their potential to spread and affect human and animal health at the local and global levels [6]. Allison White in 2009 demonstrated that antimicrobial resistance is a rapidly escalating global threat driven by the interconnected transmission of resistance genes across human, animal, and environmental systems [7].

Out of many potentially AMR microorganisms, *E. coli*, *Salmonella*, and *S. aureus* are the foremost threat-posing organisms in livestock industries [8]. *E. coli* is the main culprit in AMR list and has become the primary indicator to detect AMR. Recent investigation shows that resistance in *E. coli* increases in chicken rose from 0.15 to 0.41 between 2000 and 2018, which is an alarming situation [9]. Similarly, in *Salmonella*, surveillance showed 100% resistance to amoxicillin and erythromycin [10]. However, some regions report higher susceptibility; for example, a 2024 review found 87.5% of farm-sampled *Salmonella* strains were susceptible to all tested antibiotics, with no major resistance to priority quinolones or cephalosporins [11]. Resistance profiles for *S. aureus* vary significantly by host: swine isolates often show the highest overall resistance (e.g., 100% to tetracycline and 88% to penicillin) [12]. Methicillin-resistant *S. aureus* (MRSA) remains a critical surveillance target (*mecA*) due to its zoonotic potential [13]. Misuse of antibiotics and their prolonged use at sub-therapeutic concentrations exert selection pressure and may result in the emergence of resistant strains [14]. These organisms then spread in poultry and environments, threatening both public health and food safety [15].

Pathogenic relationship and lineage recognition are key parameters in AMR analysis. Antibiotic-sensitive organisms are continuously transformed into antibiotic-resistant ones; so, their genomic analysis is very important to understand the shift and drift in their genetic makeup through core molecular practices [16].

This study focuses on the isolation, identification, and prevalence of antimicrobial-resistant pathogens in livestock (poultry, small and large animals) and their consequences on animal farms. These pathogens or antimicrobial genes could eventually be transferred to human beings and may cause life-threatening diseases; that is why our focus was on the genetic analysis of the above-mentioned organisms.

## 2. Results

### 2.1. Common Bacterial Pathogens in Animals

A total of 536 samples were collected from various districts of Sindh Province. Most of the samples were obtained from dead animals 425 (79.29%), particularly chicken (390), goat (23), and sheep (12), whereas 111 samples from chicken (82), birds (2), cows (21), buffaloes (4), and goat (2) were taken from live animals (some slaughtered after arrival for sample collection) (Table 1). These pathological samples were obtained after postmortem examination or from live animals through various swabs, secretions, or blood samples (Table 2). All samples were processed for diagnostic purposes for the detection of disease-causing bacteria such as *S. aureus*, *E. coli*, *Salmonella*, *Klebsiella* sp., *Shigella* sp., *Pseudomonas* sp., *Streptococcus* sp., *Mycoplasma* sp., and *Pasteurella* sp. Due to ever-changing resistance patterns and current obstacles in treatment, *Salmonella* sp., *Escherichia coli*, and *Staphylococcus aureus* were selected for this antibiogram study.

### 2.2. Prevalence of Pathogens in the Samples

**Figure 1:**
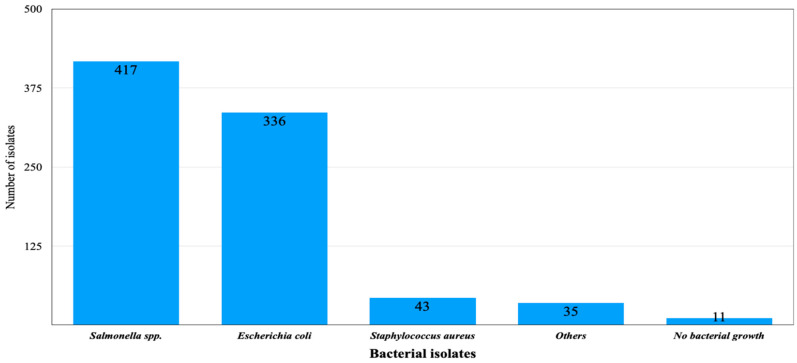
Bacterial isolates from different livestock samples.

A total of 831 isolates were obtained from 536 samples, from different animals, were analyzed for the detection of bacterial pathogens. Most of the pathogens isolated were *Salmonella* spp. 417/831 (50.18%), followed by *E. coli* 336/831 (40.43%) and *S. aureus* 43/831 (5.17%). No growth was seen in 11 samples; however, 35/831 (4.21%) samples showed growth of different bacteria other than *Salmonella*, *E. coli*, and *S. aureus* (Figure 1).

This study shows that the *Salmonella* spp. is the most common cause of bacterial infection in livestock (77.79%), followed by *Escherichia coli* (62.69%) and *Staphylococcus aureus* (8.02%). All the buffalo (4) and sheep (12) samples tested were found to be positive for *Salmonella* spp.; and the buffalo (4) and birds (chakoor and quail) were positive for *E. coli.* The percentage prevalence of *Salmonella*, *E. coli*, and *S. aureus* in the livestock is shown in (Table 3). Prevalence of these species in a large number of farm animals could be due to unhygienic conditions, contaminated water, and food supply.

### 2.3. Antibiotic-Resistant Profile in Pathogens Isolated from Animals

Antibiotic-resistant profile was determined against the most common pathogens isolated from animals, mainly poultry. Eighteen antibiotics were used to determine antibiotic-resistant profiles in the isolated organisms. Among these antibiotics, florfenicol, flumequine, and enrofloxacin are commonly used against bacterial infections in domestic and farming animals, like cows and poultry; in aquaculture; and others [17]. However, farmers and veterinarians also use most broad-spectrum antibiotics, recommended in human infections.

Around 796/831 isolates of *Salmonella* spp., *E. coli*, and *S. aureus* were screened for their antimicrobial resistance profile against a set of antibiotics, commonly used to treat bacterial infections or as growth promoters, mainly in poultry. Unfortunately, antibiotic resistance was observed against all the antibiotics tested.

#### 2.3.1. Antibiotic-Resistant Profiles of *Salmonella* spp.

**Figure 2:**
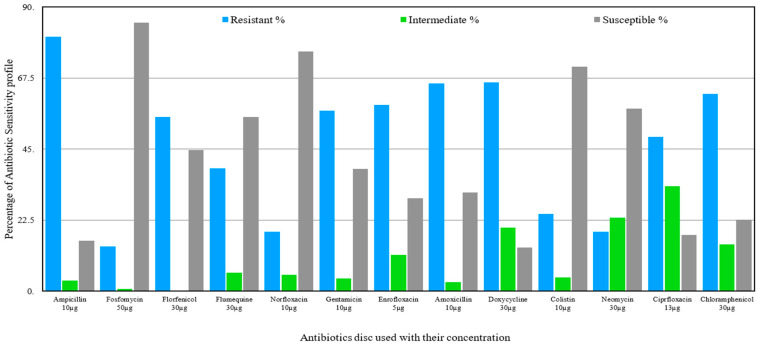
Antibiotic-resistant profile of *Salmonella* isolated from livestock.

A large number of *Salmonella* spp. isolates were found resistant to ampicillin (80.67%), doxycycline (66.19%), and amoxicillin (65.87%). Chloramphenicol, which was once very effective against *Salmonella*, has now become ineffective against 62.58% isolates. Furthermore, resistance was also observed against florfenicol (55.19%), gentamicin (57.11%), and enrofloxacin (59%). However, some of the antibiotics, like fosfomycin, norfloxacin, colistin, neomycin, and florfenicol, were found effective against 85.135%, 75.93%, 71.2%, 57.89%, and 55.19% of isolates, respectively (Figure 2).

#### 2.3.2. Antibiotic-Resistant Profiles of *Escherichia coli*

**Figure 3:**
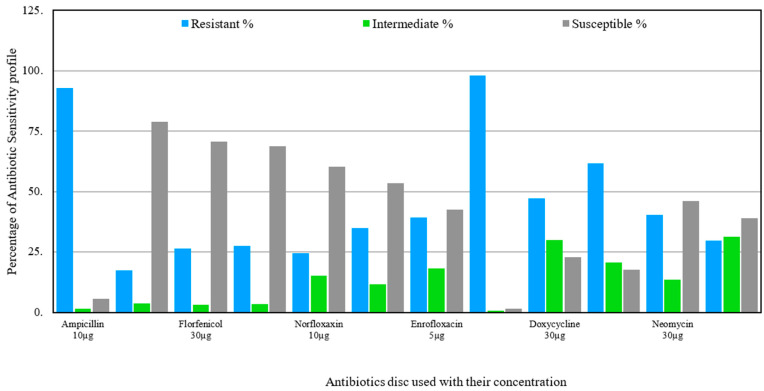
Antibiotic-resistant profile of *Escherichia coli* isolated from livestock.

Similarly, almost all the *E. coli* isolates were found resistant to amoxicillin (97.87%), ampicillin (92.75%), and oxytetracycline (61.7%). Furthermore, doxycycline (47.27%), neomycin (40.43%), enrofloxacin (39.16%), and nitrofurantoin (29.79%) were almost ineffective. However, fosfomycin, florfenicol, flumequine, norfloxacin, and gentamicin were found to be a little effective, 78.97%, 70.55%, 68.88%, 60.40%, and 53.57%, respectively, against *E. coli* isolates (Figure 3).

#### 2.3.3. Antibiotic-Resistant Profiles of *Staphylococcus aureus*

**Figure 4:**
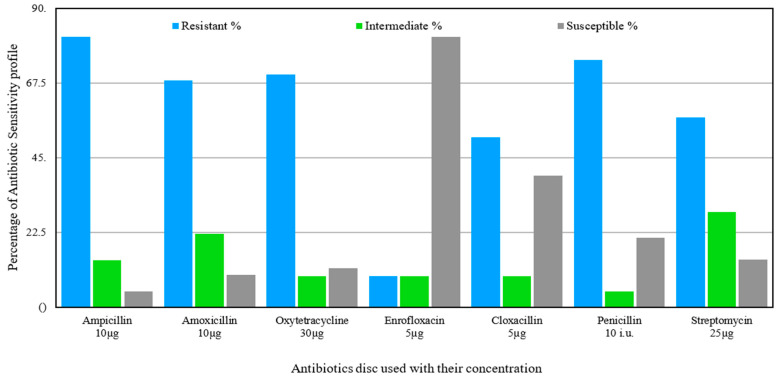
Antibiotic-resistant pattern of *Staphylococcus aureus* isolated from livestock.

Antibiotic resistance was also observed in Gram-positive *Staphylococcus aureus*. A high percentage of resistance was observed against ampicillin (81.4% isolates). Similarly, 74.42%, 70%, and 68% were found resistant to penicillin, oxytetracycline, and amoxicillin, respectively. However, enrofloxacin was the most effective against 81.4% isolates (Figure 4).

### 2.4. Multi-Drug-Resistant Strains

Multi-drug-resistant (MDR) bacteria are resistant to three or more classes of antimicrobial drugs, making treatment difficult. The prevalence of MDR has become a serious problem to treat humans and animals. The Multiple Antibiotic Resistance Index (MARI) is widely used as a surveillance tool for monitoring the prevalence of multi-drug-resistant (MDR) strains and the evaluation of appropriate treatment, particularly with *Salmonella*, *E. coli*, and *S. aureus* strains. MARI was found to be 79.7% of *Salmonella*, including all MDR strains (*n* = 332); 55.59 of *E. coli* (*n* = 189); and 88.37% of *S. aureus* (*n* = 39) with indexes greater than 0.2.

Many *Salmonella* spp., *Escherichia coli*, and *Staphylococcus aureus* were isolated from dead animals. These isolates were also found to be resistant to multiple drugs, showing the presence of multi-drug-resistant strains in the environment. Surprisingly, a high percentage of AMR was observed in almost all the strains tested. However, only 1–2% isolates were found sensitive to all the antibiotics tested.

**Figure 5:**
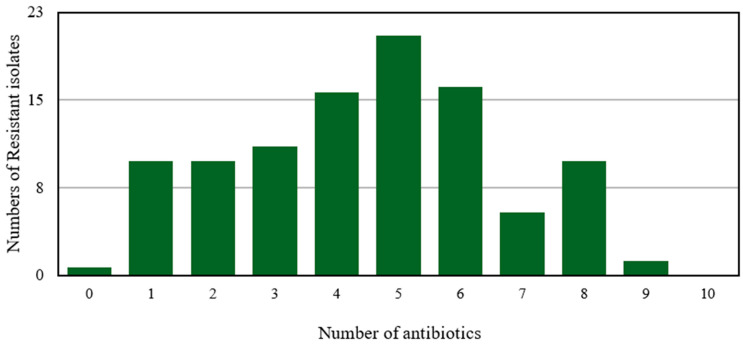
Multi-drug-resistant *Salmonella* isolates’ pattern against a number of different antibiotics.

Around 417 strains of *Salmonella* spp. were isolated and screened for the AMR profile. A high percentage of resistance was observed in 99% isolates. Surprisingly, only three isolates were found sensitive to all the antibiotics used. However, 1.22% isolates were found resistant to 90% antibiotics used for screening. Norfloxacin and colistin were found effective against some MDR strains tested (Figure 5).

**Figure 6:**
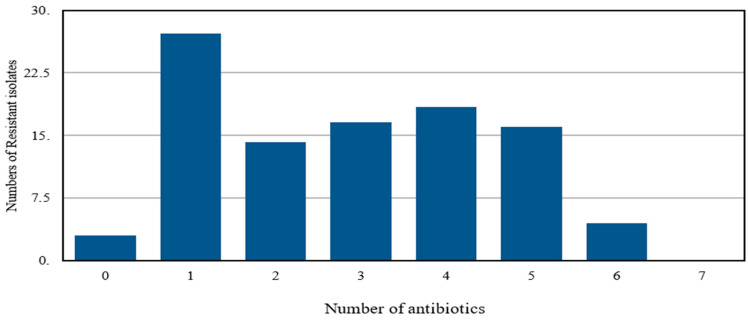
Multi-drug-resistant *Escherichia coli* isolates tested against a number of different antibiotics.

Around 336 strains of *E. coli* were isolated from poultry and large animals, mostly dead. Most of the isolates were resistant to multiple antibiotics tested. Only 4% isolates were sensitive to all the antibiotics tested. However, the majority (18.43%) of the isolates were resistant to four antibiotics and 4.53% of isolates to 90% antibiotics tested. However, no strain was found resistant to all the antibiotics tested (Figure 6).

**Figure 7:**
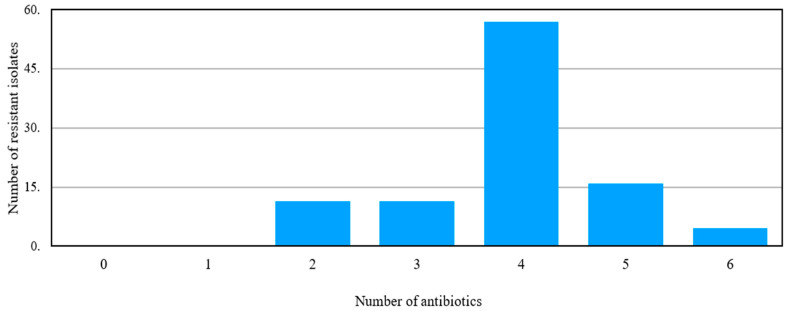
Multi-drug-resistant *Staphylococcus aureus* isolates tested against a number of different antibiotics.

Around 43 strains of *S. aureus* were isolated from poultry and large animals, mostly dead. Most of the isolates were resistant to multiple antibiotics tested. Surprisingly, no isolate was found sensitive to the antibiotics tested. However, the majority (56.82%) of the isolates were found resistant to four antibiotics used. Two isolates were found resistant to all the antibiotics tested (Figure 7).

### 2.5. Molecular Identification

**Figure 8:**
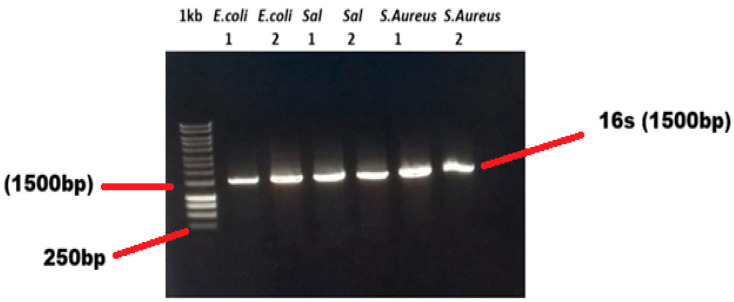
16S bacterial identification through universal primers. Well# 01 contains 1 kb reference ladder, 1500 bp bands observed in wells 2 and 3 (*E. coli)*, wells 4 and 5 (*Salmonella*), and wells 6 and 7 (*S. aureus*).

Molecular identification through PCR by 16S rRNA bacterial identification was performed randomly by selecting 14 isolates from three targeted organisms. Positive samples (Figure 8) were sent for Sanger sequencing and sequenced data are uploaded in NCBI with access numbers PX473019, PX473020, PX473021, PX473022, PX473023, PX473024, PX473025, PX473026, PX473027, PX473028, PX473029, PX473030, PX473031, and PX473032 (Table 4).

### 2.6. Phylogenetic Analysis

#### 2.6.1. *E. coli*

**Figure 9:**
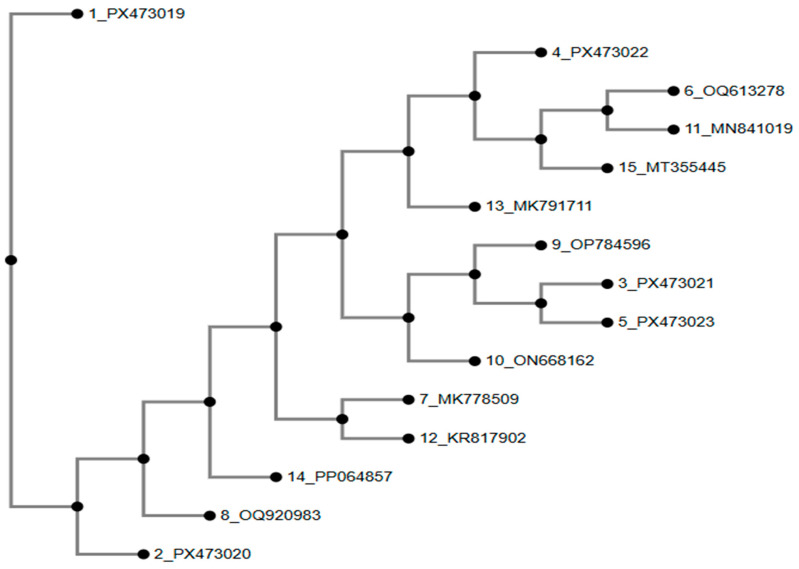
Phylogenetic analysis of *E. coli* isolates based on 16S rRNA sequences. Method of tree constriction was NJ (neighbor-joining); bootstrap resampling was 100 and model of phylogenetic analysis was Jukes–Cantor.

Phylogenetic reconstruction based on 16S rRNA gene sequences revealed that four study isolates (PX473020–PX473023) clustered closely with previously reported Pakistani *E. coli* strains (MN841019, MT355445, KR817902, MK791711, MK778509, ON668162, OP784596, OQ920983, and PP064857), which have been isolated from food, water, poultry, and clinical sources across Pakistan (Figure 9) [18,19,20].

#### 2.6.2. *Salmonella*

**Figure 10:**
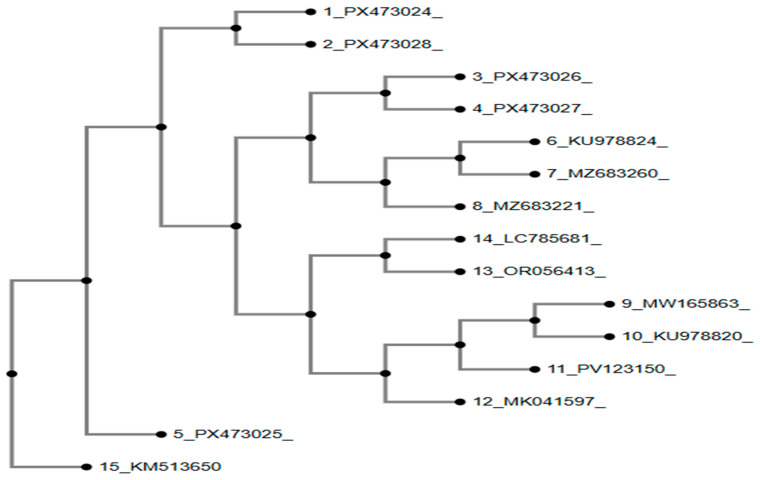
Phylogenetic analysis of *Salmonella* isolates based on 16S rRNA sequences. Method of tree constriction was NJ (neighbor-joining); bootstrap resampling was 100 and model of phylogenetic analysis was Jukes–Cantor.

Phylogenetic reconstruction of the *Salmonella* isolates based on 16S rRNA gene sequences (Figure 10) demonstrated that the study isolates (PX473029–PX473032) formed a well-supported and tightly clustered group with previously reported regional *Salmonella* reference strains, indicating a strong evolutionary relationship with circulating lineages.

#### 2.6.3. *S. aureus*

**Figure 11:**
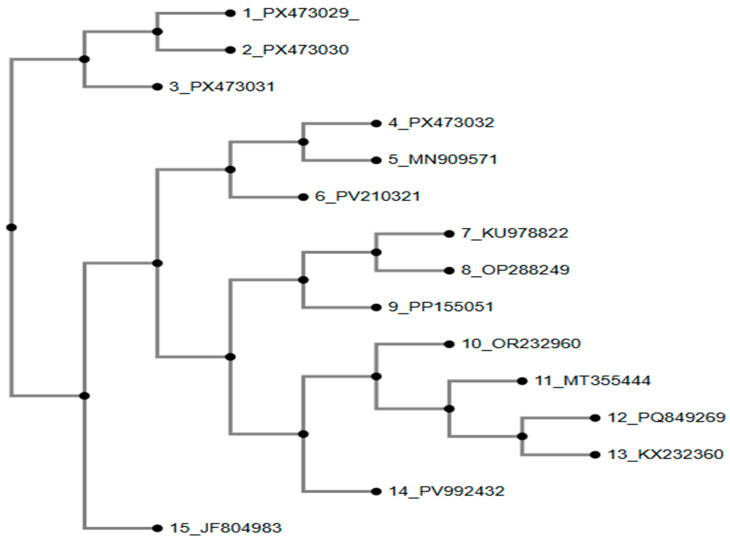
Phylogenetic analysis of *S.aureus* isolates based on 16S rRNA sequences. Method of tree constriction was NJ (neighbor-joining); bootstrap resampling was 100 and model of phylogenetic analysis was Jukes–Cantor.

Phylogenetic analysis (Figure 11) revealed that three *S. aureus* study isolates (PX473029, PX473030, and PX473031) clustered together within a well-supported clade, indicating a close evolutionary relationship and suggesting their origin from a common, actively circulating *S. aureus* lineage in the region [21].

### 2.7. Statistical Analysis

Resistance to ampicillin, gentamicin, florfenicol, flumequine, norfloxacin, amoxicillin, oxytetracycline, doxycycline, neomycin, nitrofurantoin, and enrofloxacin was significantly higher compared to that to fosfomycin (Z = 2.4–15.6, *p* < 0.05), highlighting fosfomycin as the most effective agent against *E. coli*.

Resistance to ampicillin, florfenicol, flumequine, gentamicin, enrofloxacin, amoxicillin, doxycycline, colistin, ciprofloxacin, and chloramphenicol was significantly higher compared to that to fosfomycin (Z = 2.4–16.7, *p* < 0.05). No statistically significant difference was observed between fosfomycin and norfloxacin or neomycin (*p* > 0.05) for *Salmonella*, whereas resistance to ampicillin, amoxicillin, oxytetracycline, cloxacillin, penicillin, and streptomycin was significantly higher compared to enrofloxacin (Z = 3.57–7.19, *p* < 0.05), indicating superior in vitro activity of enrofloxacin against *S. aureus*. (Supplementary Material Table).

## 3. Discussion

Antibiotics are substances of microbial origin and are used to kill pathogens. However, indiscriminate use often results in the development of resistant strains, sometimes against multiple drugs, called MDR strains [22]. However, it is important to identify resistant patterns in the pathogens and develop strategies to overcome the challenges of MDR infections [23].

Antibiotics are considered extremely essential in controlling bacterial infections; however, the emergence of antimicrobial resistance (AMR) is becoming a major public health concern worldwide. Failure of antibiotic therapy often requires the use of more toxic medications and results in increased mortality. Around 5 million deaths worldwide were associated with drug-resistant bacteria. However, nearly 90% deaths occurred in under-resourced countries [24]. Pakistan is also facing drug-resistant problems and more deaths due to antibiotic-resistant strains, particularly of *Salmonella*, *Staphylococcus*, *Enterobacterales*, and others that are included in the WHO list of “Priority pathogens” [25]. An extensively drug-resistant *Salmonella* (XDR typhoid) strain was identified that was resistant to ampicillin, cotrimoxazole, quinolone, chloramphenicol, and third-generation cephalosporins [26].

A large number of antimicrobials are used in livestock production. Around 70% of antibiotics are used worldwide to prevent diseases, promote growth, and increase feed production [27]. Mohsin et al. (2019) reported that nearly 568 tons annually, greater than China, were used in Pakistan for broiler chickens alone [28]. A survey of antibiotics used in 16 breeders shows that 88% used antibiotics for disease prevention (43%), increasing the shelf life of products (43%), disease control (21%), and at the time of breeding (21%). Furthermore, a large proportion (75–90%) of active antibiotics is excreted into the environment without being metabolized [29].

Sajid (2025) [30] reported significant resistance levels among *E. coli*, *Salmonella*, and *S. aureus* infections, with *S. aureus* in dairy cattle exhibiting the highest resistance at 88% and *E. coli* in poultry also displaying considerable rates at 87%. Survey findings revealed that 68% of respondents used antibiotics and indicated significant resistance levels among all three infections [30].

AMR bacteria from livestock can be transferred to humans through food, water, or direct contact with animals. The One Health approach is required to maintain the health of people, animals, and ecosystems. It can help to take suitable measures for prevention, detection, preparedness, response, and management—and contribute to global health security. Some antimicrobials are commonly used in humans, animals, and plants. However, there are some separate classes of antimicrobials for humans (carbapenems) and animal (flavophospholipol and ionophores) [31].

Development of AMR must be observed at national levels by monitoring and improving facilities in health care systems to reduce the cost of treatment and prolong hospitalization. Furthermore, implementation of policies is needed to control the use of antimicrobials, providing clean water, improving sanitation, and hygiene for humans and animals. Moreover, public awareness and knowledge about the consequences of AMR organisms and irregularities with legislation should be raised [32]. AMR should be managed from a “One Health” perspective. Global efforts are required to make significant progress in controlling AMR. This may be put on the international political agenda and address the One Health approach to control life-threatening infections [33].

The antimicrobial resistance profiles identified in the present investigation reflect trends increasingly reported across South Asian veterinary settings, where extensive and often empirical antimicrobial usage has contributed to reduced therapeutic efficacy. In *E. coli*, fosfomycin exhibited the lowest resistance frequency, while ampicillin and amoxicillin showed significantly elevated resistance levels (*p* < 0.05). Comparable resistance to β-lactam antibiotics has been documented in regional studies; for instance, Haulisah et al. reported substantial resistance of *E. coli* isolates from food animals to penicillin-class drugs, with resistance rates exceeding 77% [34]. Similarly, Ahmed et al. observed high resistance of *E. coli* to ampicillin (79.7%) in meat-associated isolates, further indicating the diminished effectiveness of commonly used β-lactams in animal production systems within the region [35]. The relatively low resistance to fosfomycin observed in our study suggests limited exposure and preserved activity, positioning it as a potentially effective alternative for treating *E. coli* infections in veterinary practice.

In *Salmonella* isolates, fosfomycin and norfloxacin demonstrated comparatively lower resistance rates (14.9% and 19.9%, respectively), whereas doxycycline and ampicillin showed higher resistance. These findings are partially consistent with regional surveillance data reporting substantial resistance of *Salmonella* spp. to tetracycline-class antibiotics, including oxytetracycline [36]. Variations in resistance profiles across studies may reflect differences in antimicrobial usage patterns, animal species, and geographic distribution, which are known to influence selective pressure in South Asian livestock production systems [37].

For *S. aureus*, enrofloxacin showed the lowest resistance (18.6%) in the present study, while significantly higher resistance was observed for other tested antibiotics (*p* < 0.05). This pattern aligns with previous veterinary investigations reporting high resistance of *S. aureus* to ampicillin, neomycin, and streptomycin [38]. Furthermore, the emergence of multi-drug-resistant and pan-drug-resistant *S. aureus* strains, as reported by Panchal et al., underscores the escalating therapeutic challenges associated with staphylococcal infections in animal populations [39]. Although some studies have reported moderate susceptibility of *E. coli* and *Salmonella* to selected antimicrobials [40], the overall evidence indicates a consistent decline in susceptibility to frequently administered antibiotics across South Asian veterinary settings [41].

Taken together, these findings reinforce that resistance to β-lactams and tetracyclines remains widespread among major veterinary pathogens, including *E. coli*, *Salmonella*, and *S. aureus*, while agents such as fosfomycin and fluoroquinolones may still retain comparatively lower resistance. Continuous monitoring of resistance trends and judicious antimicrobial selection are therefore critical to preserving the efficacy of remaining therapeutic options in the region.

Phylogenetic analysis revealed that most study isolates clustered closely with previously reported Pakistani reference strains, indicating the persistence of dominant bacterial lineages circulating across the region [42]. *Escherichia coli* isolates PX473020–PX473023 grouped within well-supported local clades, while PX473019 formed a distinct branch, suggesting lineage-level heterogeneity likely shaped by localized evolutionary pressures. *Salmonella* isolates (PX473029–PX473032) showed tight clustering with regional reference strains, reflecting limited genomic divergence and continued circulation of established poultry-associated lineages in South Asia [43]. Minor intra-clade variations indicate ongoing microevolution within otherwise stable populations. Overall, these patterns underscore the coexistence of dominant and diverse bacterial lineages and highlight the importance of continued molecular surveillance for tracking pathogen evolution and antimicrobial resistance [44].

In developed countries, the use and sales of antibiotics in animals are effectively controlled and switched to alternative methods like the use of effective vaccines. Antimicrobials are widely used in most underdeveloped and developing countries in large quantities in the animal food industry [45]. Arshed et al. reported significant AMR in food animals in Pakistan, highlighting high resistance in E. coli and Salmonella isolates [46]. However, a report shows that, in European countries, the average consumption of antimicrobials is significantly reduced in food-producing animals than in the human sector [18].

Vaccine use has been proven to control highly infectious diseases. Recently, a pandemic was controlled successfully using vaccines that proved to be safe and reliable in controlling COVID-19. Efforts may be made to develop effective vaccines against common pathogens and their use as reliable alternatives. Furthermore, other methods may be explored as alternatives to antimicrobials, such as phages, probiotics, antibodies, etc.

## 4. Material and Methods

Farmers are facing microbial threats in the form of high morbidity and mortality, especially in poultry. This study was designed to identify the most common pathogens and their resistance patterns to find the most suitable available drug to control infections. Most of the animals (poultry) brought to the SIAH for diagnosis were dead or died before reaching the diagnostic lab (79.29%).

### 4.1. Study Area

This study was conducted in one of the largest and pioneer institutes of animal health and surveillance center of Sindh Province, Pakistan, named the Sindh Institute of Animal Health (SIAH). The Sindh Institute of Animal Health has had the privilege to serve the livestock division of Sindh province for the past 30 years.

### 4.2. Study Duration

The present study was taken place in the Antimicrobial Resistance (AMR) Laboratory at the Sindh Institute of Animal Health from January 2023 to December 2024.

### 4.3. Sample Size

Around 536 animal samples were examined for microbial infections, including chicken (472), birds (1 chakoor and 1 quail), cow (21) and buffalo (4), goat (25), and sheep (12). Most of the animals were dead or died before sampling; however, the majority of the samples were obtained from dead animals after a postmortem (79.29%) at the lab (Table 1).

### 4.4. Pathological Samples

Clinical samples were collected from live animals or dead animals after performing postmortem. Samples were collected after pathological examination by the pathologist at SIAH. Sometimes more than one sample was collected from a single animal for an appropriate diagnosis. The sample includes liver, spleen, feces, heart, trachea, swab, milk, lung, oral swab, tissues, intestine, meat, proventriculus, and kidney (Table 2).

### 4.5. Isolation and Identification

All samples were inoculated in the respective broth mediums for enrichment purposes and incubated for 24 h at 37 °C [47]. Tryptone water (Oxiod, UK) was used for feed/distilled water/saline samples; tetrathionate broth (Oxoid, UK) for fecal samples; tryptose phosphate broth (Oxoid, UK) for organ and samples; and buffered peptone water (Oxiod, UK) for milk samples.

The isolation and identification of *Salmonella* sp., *E. coli*, and *S. aureus* was carried out using selective and differential media, i.e., Mannitol Salt Agar, Oxoid, UK (MSA) for *S. aureus*, Eosin Methylene Blue Agar, Oxoid, UK (EMB) for *E*. *coli*, and Bismuth Sulfide agar, Oxoid, UK (BSA) for *Salmonella* sp. The enriched samples were then streaked on the aforementioned culture plates and incubated at 37 °C (witeg Wisd WIF-105, Germany) for 24 h [48]. Biochemical tests were performed using API kits (Biomerieux, France) for confirmation of isolates. The tests were performed in triplicate to exclude false negative results. And they were compared with the available ATCC strains of the respective bacteria.

### 4.6. Antimicrobial Sensitivity Test

Antimicrobial resistance (AMR) was determined using the Kirby–Bauer disc diffusion method [49]. All the purified bacterial isolates were grown in the nutrient broth (Oxoid, UK), and growth was adjusted to 0.5 McFarland, i.e., 1–2 × 10^8^ CFU/ml. Bacterial lawn of each isolate was prepared on Muller–Hinton agar, (Oxoid, UK) plates. The antibiotic discs were placed on the prepared lawn of each organism, and the plates were incubated at 37 °C (witeg Wisd WIF-155, Germany) for 24 h. Zones of growth inhibition around antibiotic discs were measured by vernier calipers to compare with standard zones as described in Clinical and Laboratory Standard Institute, 2025 (CSLI) [50]. The panel of antibiotic disks (Oxoid, UK) used against *Salmonella* spp. includes ampicillin (10 µg), doxycycline (30 µg), chloramphenicol (30 µg), florfenicol (30 µg), enrofloxacin (5 µg), and norfloxacin (10 µg); for *E. coli*, the antibiotic panel was ampicillin (10 µg), doxycycline (30 µg), neomycin (30 µg), enrofloxacin (5 µg), florfenicol (30 µg), and norfloxacin (10 µg); and the antibiotic panel used for *S. aureus* was ampicillin (10 µg), penicillin (10 iu), oxytetracycline (30 µg), amoxicillin (10 µg), enrofloxacin (5 µg), cloxacillin (5 µg), and streptomycin (25 µg). These panels of antibiotics were derived from the latest edition of CLSI [51].

Among these antibiotics, florfenicol, flumequine, and enrofloxacin are commonly used against bacterial infections in domestic and farming animals, like cows and poultry; in aquaculture; and others. However, farmers and veterinarians also use most broad-spectrum antibiotics, recommended for human infections [46].

### 4.7. Multiple Antibiotic Resistance Index (MARI)

The MARI was calculated by the method described by Krumperman (1983) [52] using the following equation: Multiple Antibiotic Resistance Index = *a*/*b*, where *a* represents the number of antibiotics to which the isolate was resistant and *b* represents the total number of antibiotics against which the isolate was tested. A MARI > 0.2 indicates the existence of an isolate from high-risk contaminated sources with the frequent use of antibiotics, whereas values ≤ 0.2 show that bacteria are from sources that were exposed to less antibiotic usage [53].

### 4.8. Molecular Identification

16S bacterial identification of 14 samples (5 *E. coli*, 5 *Salmonella*, and 4 *S. aureus*) were performed using 16S universal primers 27F 5′-AGA GTT TGA TCC TGG CTC AG-3′ and 1492R 5′-GGT TAC CTT GTT ACG ACT T-3′ with thermal cyclic (Turbocycler Model # TCST-9612.TCST-9622, Blue-Ray Biotech Taipei City, Taiwan) condition with one cycle of initial denaturation at 94 °C for 5 min and max 40 cycles at 94 °C for 30 s annealing at 49.5 °C for 30 s and extension 72 °C for 2 min, final extension for 1 cycle at 72 °C for 10 min [54]. PCR results were visualized through 1% Agarose Gel electrophoresis (BIO-RAD, Model # PowerPac 300, California, USA). PCR positive samples were sent for sequencing to Macrogen Korea.

### 4.9. Statistical Analysis

A two-proportion Z-test was applied to compare antimicrobial resistance patterns of *Escherichia coli*, *Salmonella*, and *S. aureus* using compiled data from the 2023–2024 period (*n* = 336 for *E. coli*, *n* = 417 for *Salmonella*, and *n* = 43 for *S. aureus*). Resistant and intermediate isolates were merged for analysis [55]. Statistical comparisons were conducted using a two-tailed test at a 95% confidence interval (α = 0.05), with a total of 336 (for *E. coli*), 417 (for *Salmonella*), and 43 (for *S. aureus)* isolates analyzed per antibiotic.

## 5. Conclusions

Microorganisms are rapidly becoming resistant against a variety of antibiotics. This shift is not only reported in humans, but also these types of resistance are notably identified in livestock as well. Prominent microorganisms like E. coli, Salmonella, and S. aureus are included in this study, which are a major concern of “One Health”. An increasing trend of resistance was observed in all the three microorganisms, and this study will be a cornerstone for the researcher to focus on AMR in livestock as well. Additional molecular analysis of resistance genes is now planned to further investigate evidence of overlapping patterns of transferable resistance genes between livestock and soil.

Proactive strategies should be planned to stop this increasing trend of AMR in livestock by providing awareness sessions to all farmers who unintentionally administrate unnecessary antibiotics without DVM consideration.

## Figures and Tables

**Table 1:** The number of animals examined for the presence of microbial infections.

Animal Species	Number of Animals	Live Animals	Dead Animals
Chicken	472	82	390
Birds (chakoor and quail)	2	2	0
Cows	21	21	0
Buffalo	4	4	0
Goat	25	2	23
Sheep	12	0	12
Total of Samples	536	111 (20.71%)	425 (79.29%)

**Table 2:** Pathological samples collected from different animals for the detection of pathogens.

**Collected Samples**	**Sample Matrices**	**Liver**	**Spleen**	**Heart**	**Trachea**	**Lungs**	**Kidney**	**Proventriculus**	**Cloacal**	**Feces**	**Milk**	**Swab**	**Oral**	**Tissues**	**Intestine**	**Meat**	**Total**
	**Animal Species**	**Chickens and Birds**									**Cow, Buffaloes, Sheep, and Goat**			**Cow and Buffaloes**			
	2023	73	23	6	54	34	3	2	14	2	12	14	13	6	2	0	258
	2024	81	17	6	60	39	0	6	20	2	13	18	7	6	2	1	278
	Total	160	40	12	114	73	3	8	34	4	25	32	20	12	4	1	536

**Table 3:** Percentage of prevalence of *E. coli*, *Salmonella*, and *S. aureus* in livestock.

Animal (Number of Samples)	*E. coli*	*Salmonella*	*S. aureus*
Cow (21)	76.19%	85.71%	14.29%
Buffalo (4)	100%	100%	0%
Sheep (12)	66.67%	100%	0%
Goat (25)	68%	68%	12%
Poultry (472)	61.23%	77.54%	6.99%
Birds (2)	100%	-	-
Total (536)	62.69%	77.79%	8.02%
Multiple Antibiotic Resistance Index	55.59%	79.7%	88.37%

**Table 4:** Accession numbers submitted to NCBI with the respective organisms.

S. No.	Accession Number	Organism
1	PX473019	*E. coli*
2	PX473020	
3	PX473021	
4	PX473022	
5	PX473023	
6	PX473024	*Salmonella* spp.
7	PX473025	
8	PX473026	
9	PX473027	
10	PX473028	
11	PX473029	*Staphylococcus aureus*
12	PX473030	
13	PX473031	
14	PX473032	

## Data Availability

The datasets generated for this study are available upon request from the corresponding author.

## Supplementary Materials

The following supporting information can be downloaded at: https://www.mdpi.com/article/10.3390/antibiotics15050461/s1.
